# A Novel Splicing Variant of Peroxisome Proliferator-Activated Receptor-γ (*Pparγ1sv*) Cooperatively Regulates Adipocyte Differentiation with *Pparγ2*

**DOI:** 10.1371/journal.pone.0065583

**Published:** 2013-06-19

**Authors:** Yasuhiro Takenaka, Ikuo Inoue, Takanari Nakano, Yuichi Shinoda, Masaaki Ikeda, Takuya Awata, Shigehiro Katayama

**Affiliations:** 1 Department of Diabetes and Endocrinology, Saitama Medical University, Saitama, Japan; 2 Department of Biochemistry, Saitama Medical University, Saitama, Japan; 3 Misato Central General Hospital, Saitama, Japan; 4 Department of Physiology, Saitama Medical University, Saitama, Japan; Baylor College of Medicine, United States of America

## Abstract

Peroxisome proliferator-activated receptors (PPARs) are nuclear receptors that regulate expression of a number of genes associated with the cellular differentiation and development. Here, we show the abundant and ubiquitous expression of a newly identified splicing variant of mouse *Pparγ* (*Pparγ1sv*) that encodes PPARγ1 protein, and its importance in adipogenesis. The novel splicing variant has a unique 5′-UTR sequence, relative to those of *Pparγ1* and *Pparγ2* mRNAs, indicating the presence of a novel transcriptional initiation site and promoter for *Pparγ* expression. *Pparγ1sv* was highly expressed in the white and brown adipose tissues at levels comparable to *Pparγ2*. *Pparγ1sv* was synergistically up-regulated with *Pparγ2* during adipocyte differentiation of 3T3-L1 cells and mouse primary cultured preadipocytes. Inhibition of *Pparγ1sv* by specific siRNAs completely abolished the induced adipogenesis in 3T3-L1 cells. C/EBPβ and C/EBPδ activated both the *Pparγ1sv* and *Pparγ2* promoters in 3T3-L1 preadipocytes. These findings suggest that *Pparγ1sv* and *Pparγ2* synergistically regulate the early stage of the adipocyte differentiation.

## Introduction

Obesity has become a growing worldwide health problem in recent years. An excessive accumulation of white adipose tissue caused by increases in the cell number and size of newly differentiated white adipocytes from preadipocytes is a major cause of obesity. Thus, the elucidation of mechanisms of adipocyte differentiation is essential for understanding the pathogenesis of obesity and obesity-associated diseases.

3T3-L1, a cell line derived from mouse 3T3 fibroblast, has been widely used as a model of adipocyte differentiation [1]. The addition of chemicals and hormones such as dexamethasone or insulin into culture media of 3T3-L1 cells induces the synthesis and accumulation of intracellular triglycerides and changes in their morphology from fibroblast-like to adipocyte-like [2]. During the progression, a number of adipocyte-related genes are up-regulated by a sequential induction of transcription factors such as peroxisome proliferator-activated receptor γ (PPARγ) and members of the CCAAT/enhancer-binding proteins (C/EBPα, C/EBPβ, and C/EBPδ) [3]. PPARγ is a member of the ligand-dependent nuclear receptor superfamily and plays a pivotal role in adipogenesis and intracellular lipid accumulation. C/EBPs belong to a family of the basic region-leucine zipper (bZIP) transcription factors. C/EBPβ and C/EBPδ are transiently expressed very early during adipocyte differentiation [4], which in turn transactivate gene expression of PPARγ and C/EBPα [5]. Both proteins cooperatively promote downstream adipocyte-related genes such as the adipocyte-specific fatty acid-binding protein gene (FABP4) to develop functional adipocytes.

PPARγ is expressed as at least two splicing variants, the ubiquitously expressed *Pparγ1* and adipocyte-specific *Pparγ2* [6], [7]. PPARγ2 protein that is translated from *Pparγ2* mRNA is longer than PPARγ1 (from *Pparγ1*) by 30 amino acid residues at the N-terminus in mice. PPARγ2 protein has been considered to play a critical role in the adipogenesis, because *Pparγ2* mRNA, but not *Pparγ1*, is abundantly expressed in the adipose tissues. However, PPARγ1 expression also has been observed in adipocytes at similar level to PPARγ2 in the previous reports [8]–[10], which complicated the role of PPARγ1 in adipogenesis.

In addition to *Pparγ1* and *Pparγ2*, several unique splicing variants of *Pparγ* has been reported [11], [12]. We have recently reported a novel PPARγ splicing variant in humans that is regulated by circadian rhythmic D-site binding protein, DBP [13]. However, the involvement of this splicing variant in adipogenesis has not been uncovered.

In this paper, we report the identification of a novel *Pparγ* splicing variant, *Pparγ1sv*, in mice that is synergistically up-regulated with *Pparγ2* during adipocyte differentiation of 3T3-L1 cells and mouse primary cultured preadipocytes. Knock-down experiments using siRNA specifically targeting to *Pparγ1sv* revealed that PPARγ1 protein expressed during adipogenesis is derived from *Pparγ1sv* mRNA. Thus, this novel splicing variant could explain the induced PPARγ1 protein during adipocyte differentiation. Furthermore, knock-down of *Pparγ1sv* abolished the induced adipogenesis of 3T3-L1 cells, indicating that PPARγ1 from *Pparγ1sv* plays a crucial and synergistic role with PPARγ2 in adipogenesis.

## Materials and Methods

### Cell Culture, Differentiation, and Staining

3T3-L1 and ST2 cells were obtained from the Japan Health Science Foundation, Health Science Research Resources Bank (Osaka, Japan) and RIKEN Cell Bank (Tsukuba, Japan), respectively. Mouse primary cultured preadipocytes isolated from white adipose tissues of newborn mice were purchased from Primary Cell Co., Ltd (Hokkaido, Japan). 3T3-L1 and ST2 cells were maintained in DMEM and RPMI1640 (Life Technologies), respectively, supplemented with 10% fetal bovine serum (Sigma-Aldrich) and penicillin-streptomycin at 37°C in a humidified atmosphere of 5% CO_2_. Cells were passaged every 3 days. For adipocyte differentiation, we plated cells in 3-cm or 6-cm dishes, allowed them to grow at 95–100% confluency, and then changed the culture medium to DMEM containing 0.25 µM dexamethasone, 500 µM isobutylmethylxanthine, and 1 µM insulin. Primary preadipocytes were cultured in DMEM containing 2.5 µM dexamethasone and 10 µg/ml insulin for two days to start differentiation into adipocytes according to the manufacturer’s instructions. We estimated the adipocyte differentiation by staining intracellular lipid droplets with Oil Red O or quantifying cellular triglycerides content with AdipoRed assay reagent (Lonza).

### Cloning of a Novel Splicing variant of Mouse *Pparγ*

Total RNA was purified from adipocyte-differentiated 3T3-L1 cells (9 days after the chemical induction) using ISOGEN (Nippon Gene). 5′- and 3′-Ready SMART cDNA was synthesized from 1 µg of total RNA using the SMART RACE cDNA synthesis kit according to the manufacturer’s instructions (Takara Bio). The 5′-end of mouse *Pparγ* cDNA was amplified from 5′-Ready SMART cDNA using mPPARg_5RACE_LP2, 5′-TTGGGTCAGCTCTTGTGAATGGAATG-3′ and Universal Primer Mix (UPM) (Takara Bio). After sequencing the 5′-rapid amplification of cDNA end (RACE) product, full-length cDNA was amplified from 3′-Ready SMART cDNA using mPPARg_novel_5′term, 5′-GGGGCCTGGACCTCTGCTGGGGATCT-3′ and UPM, cloned into pGEM-T Easy vector (Promega) and sequenced.

### Quantitative RT-PCR

To quantify mouse *Pparγ1sv*, *Pparγ1*, *Pparγ2*, and 18S ribosomal RNA expression in 3T3-L1, ST2, and primary cultured cells by quantitative PCR (qPCR), we used the THUNDERBIRD SYBR qPCR mix (Toyobo) in an ABI Prism 7900 HT sequence detection system (Life Technologies). For analyses of the tissue distribution of *Pparγ* expression, a part of the cDNA was amplified from the normalized MTC Mouse Panel I and III (Takara Bio). Expression of *Pparγ1sv*, *Pparγ1*, *Pparγ2*, and 18S ribosomal RNA was also analyzed by qPCR using the first strand cDNA prepared from mouse white and brown adipose tissues, respectively (n = 4). Primer sequences used in qPCR were as follows, mPPARg_novel_5′term, 5′-GGGGCCTGGACCTCTGCTGGGGATCT-3′ and mPPARg_E1-Rv, 5′-GGCCAGAATGGCATCTCTGTGTCAA-3′ for *Pparγ1sv* cDNA; mPPARg1_Fw2, 5′-GCTGAGGGGACGGGCTGAGGAGAA-3′ and mPPARg_E1-Rv for *Pparγ1* cDNA; mPPARg2_Fw, 5′-GTTATGGGTGAAACTCTGGGAGAT-3′ and mPPARg_E1-Rv for *Pparγ2* cDNA; LEM-m18S-F, 5′-CGGCTACCACATCCAAGGAA-3′ and LEM-m18S-R, 5′-GCTGGAATTACCGCGGCT-3′ for 18S ribosomal RNA.

### Plasmid Constructs and Small Interfering RNAs

For the assessment of small interfering RNA (siRNA) specificity, the full-length *Pparγ1sv* cDNA or *Pparγ2* cDNA was subcloned into *Xba* I site between the stop codon of luciferase coding region (luc+) and the poly(A) signal in pGL3-Control vector (Promega) in sense or antisense direction. For the promoter assay of mouse *Pparγ*, the 5′-flanking region of the *Pparγ1sv* (−969 to +31), *Pparγ1* (−1,529 to +31), and *Pparγ2* (−1,473 to +41) were amplified with Advantage 2 DNA polymerase (Takara Bio) from the genomic DNA of 3T3-L1 cells, and were subcloned into pGL3-Basic (Promega). The coding regions of mouse C/EBPα, C/EBPβ, and C/EBPδ were amplified by Advantage 2 DNA polymerase from the cDNA of 3T3-L1 cells, and were subcloned into pcDNA3.1(-) (Life Technologies) for overexpression in luciferase reporter experiments. All expression vectors were purified using an EndoFree plasmid maxi kit (Qiagen). siRNAs targeting mouse *Pparγ1sv*, *Pparγ2*, common sequence of *Pparγ*, and C/EBPβ mRNAs were purchased from Life Technologies (Stealth RNAi). Target sequences of mRNAs were as follows: 5′-GAUCUGAAGGCUGCAGCGCUAAAUU-3′ (siγ1sv22) and 5′-GGCUGCAGCGCUAAAUUCUUCUUAA-3′ (siγ1sv30) for *Pparγ1sv*; 5′-CCAGUGUGAAUUACAGCAAAUCUCU-3′ (siγ2_8) and 5′-GGGUGAAACUCUGGGAGAUUCUCCU-3′ (siγ2_48) for *Pparγ2*; 5′-CCAGGAGAUCUACAAGGACUUGUAU-3′ (siγcοmmοn1) and 5′-UCAAGGGUGCCAGUUUCGAUCCGUA-3′ (siγcommon2) for all transcripts of *Pparγ*; 5′-CCGCCGCCUUUAGACCCAUGGAAGU-3′ (siC/EBPβ #1) and 5′-CCCAUGGAAGUGGCCAACUUCUACU-3′ (siC/EBPβ #2) for C/EBPβ. For siRNA transfections, 5×10^5^ cells/well were seeded onto 6-well culture plates and transfected with the above siRNAs using Lipofectamine RNAiMAX (Life Technologies) according to the manufacturer’s instructions.

### Western Blotting and Antibodies

Cells cultured in 6-cm dishes were trypsinized and harvested in 1 ml phosphate buffered saline. Nuclear extracts were prepared using NE-PER nuclear and cytoplasmic extraction reagents (Thermo scientific), mixed with 5× sodium dodecyl sulfate (SDS) sample buffer containing 2-mercaptoethanol, heated at 95°C for 3 min, and then loaded onto a 12.5% SDS-polyacrylamide gel. Proteins were transferred to a PVDF membrane and incubated in 1% Western blocking reagent (Roche Applied Science) at room temperature for 1 hr. The membrane was then incubated overnight with anti-PPARγ (A3409A) (Perseus Proteomics), anti-C/EBPβ (Santa Cruz Biotechnology), anti-C/EBPα (Santa Cruz Biotechnology), anti-Lamin B1 (abcam), anti-FABP4 (Cell Signaling Technology), anti-DLK (Santa Cruz Biotechnology), or anti-α-tubulin (Sigma-Aldrich) antibody diluted in 0.5% Western blocking reagent (1∶1,000). HRP-conjugated goat anti-mouse IgG (Sigma-Aldrich) or anti-rabbit IgG (GE healthcare) antibody was used as the secondary antibody and detected with ECL prime reagent (GE Healthcare). The chemiluminescent signal was exposed to Hyperfilm ECL (GE Healthcare).

### Luciferase Reporter Assay

Preadipocyte 3T3-L1 cells in the 12-well plate were transiently transfected with 0.6 µg of promoter/luciferase reporter construct (pGL3-Basic), 0.5 µg of overexpression construct (pcDNA3.1), and 0.5 µg of the constitutive *Renilla* luciferase expression vector (pGL4.74) (Promega) for normalization in a well using Lipofectamine 2000 transfection reagent (Life Technologies). For the assessment of siRNA specificity, 3T3-L1 cells were simultaneously transfected with each of siRNAs, the reporter plasmid pGL3-Control containing the *Pparγ1sv* or *Pparγ2* cDNA, and pGL4.74 for normalization. Cells were harvested 1 or 2 days after the transfection, and luciferase assays were performed using the Dual-luciferase reporter assay system (Promega). Luminescence was counted for 10 sec using a MiniLumat LB 9506 luminometer (Berthold).

## Results

### A Novel Mouse *Pparγ* Splicing variant and its Gene Structure

**Figure 1:**
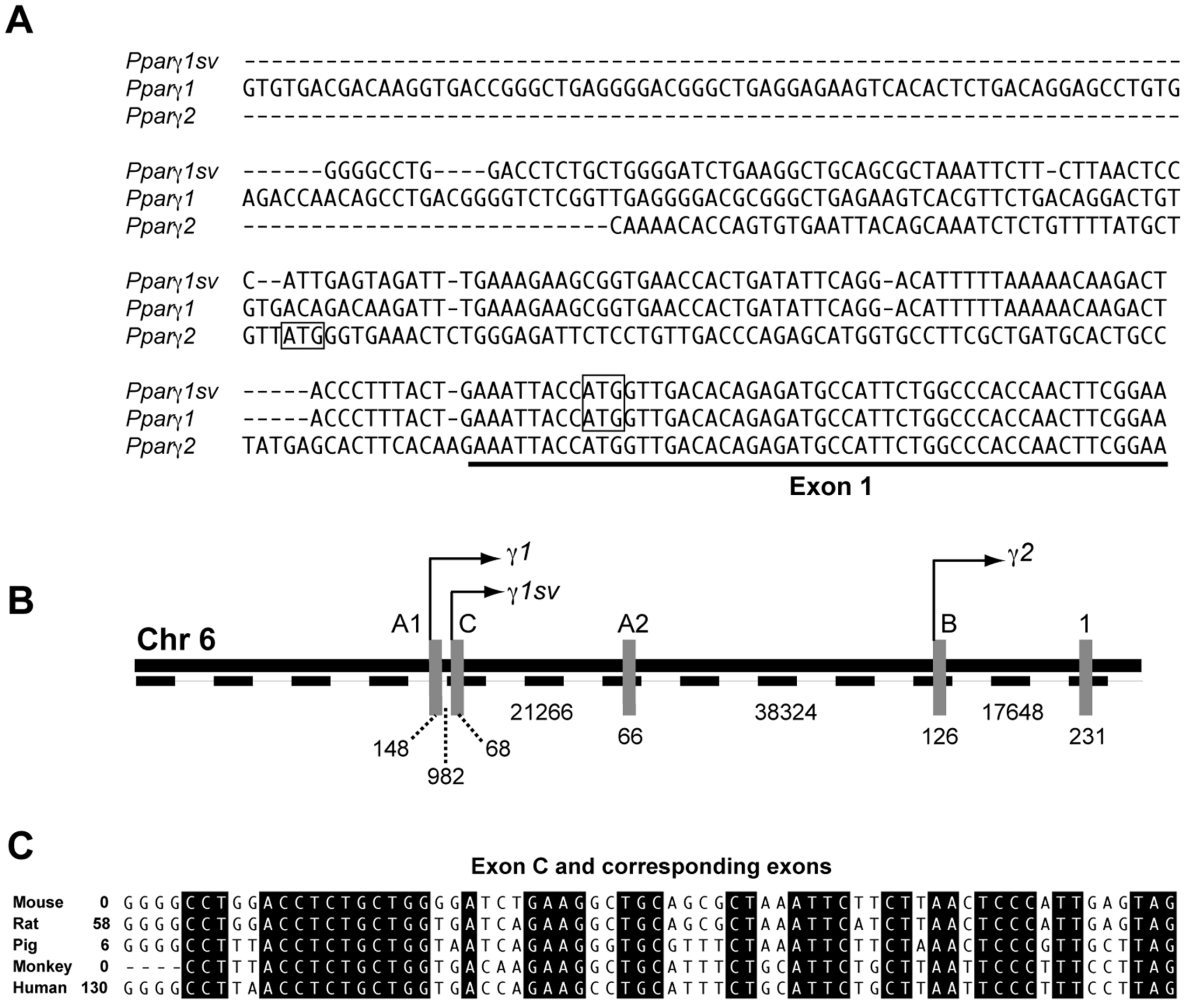
Gene and cDNA structures of the novel splicing variant of mouse *Pparγ*. (A) Alignment of 5′-end sequences of *Pparγ1sv*, *Pparγ1*, and *Pparγ2* cDNAs. Each initiation codon is outlined, and exon 1, which is common to all three, is underlined. (B) Gene structure of N-terminal exons and common exon 1 of mouse *Pparγ* on chromosome 6. Distances between two exons and exon lengths are indicated as numbers of nucleotides. Arrows indicate the positions of the transcription initiation site of each transcription variant. (C) Multiple alignment of nucleotide sequences of mouse exon C and corresponding exons in other mammals. Distances between the 5′-end of each cDNA and exon C or corresponding exons of other mammals are indicated as numbers of base pairs.

We amplified the 5′-ends of mouse *Pparγ* cDNAs using a reverse primer based on the sequence of exon 1 and a terminal adaptor primer from a cDNA library prepared from adipocyte differentiated 3T3-L1 cells (day 9). The amplified 5′-end products (∼350 bp) contained the coding sequence and 5′-UTR of mouse *Pparγ* cDNAs. We sequenced 22 clones, nine of which contained 5′-end sequences of *Pparγ2*, one contained the 5′-end of *Pparγ1*. Remaining 12 clones possessed a unique 5′-UTR that was different from those of *Pparγ1* and *Pparγ2* (Fig. 1A). Full-length cDNAs of the novel splicing variant were then amplified using the 3′-end adapter primer and 5′-end gene-specific primer that was designed based on the sequences of 5′RACE products. Sequencing of the full-length cDNA showed that the novel splicing variant encoded an identical amino acid sequence of mouse PPARγ1. We have designated this novel splicing variant as *Pparγ1sv*. The complete sequence of *Pparγ1sv* cDNA was deposited in the DDBJ/EMBL/GenBank database under the accession number AB644275. The transcription initiation site of the novel splicing variant was located on the novel exon C (68 bp) by aligning its sequence with the mouse *Pparγ* genomic sequence on chromosome 6 (Fig. 1B). Exon C is located far (∼60 kbp) from exon B of *Pparγ2* whereas it is relatively close (∼1 kbp) to exon A1 of *Pparγ1* (Fig. 1B). A homology search using the BLAST program revealed that exon C shared 81% sequence identity with porcine exon A’ (GenBank no. AB121691) and 77% with exon C (or A’) of human PPARγ transcript variant 3 (NM_138711). Alignment of the nucleotide sequence of mouse exon C with those of corresponding exons of other mammals are shown in Fig 1C. Additional information about mouse *Pparγ1sv* and its homologous transcription variants in other mammals is summarized in Table 1.

**Table 1:** Summary of *Pparγ1sv* and other mammalian cDNAs that contain the unique exons illustrated in Fig. 1C.

Gene name	Source	Unique exon	Accession #
*Pparγ1sv*	Mouse	C	AB644275
PPARγ1, PPARγ tv*2	Rat	ND**	AF156665, NM_001145366
PPARγ1c	Pig	A’	AB097928
PPARγ1, PPARγ6, PPARγ7	Monkey	A1	AY048694, AY048699, AY048700
PPARγ tv*3	Human	C, A’	AB472042, NM_138711

* transcription variant,

** not defined.

### Tissue Distribution and Relative Abundance of *Pparγ1sv* in Mice

**Figure 2:**
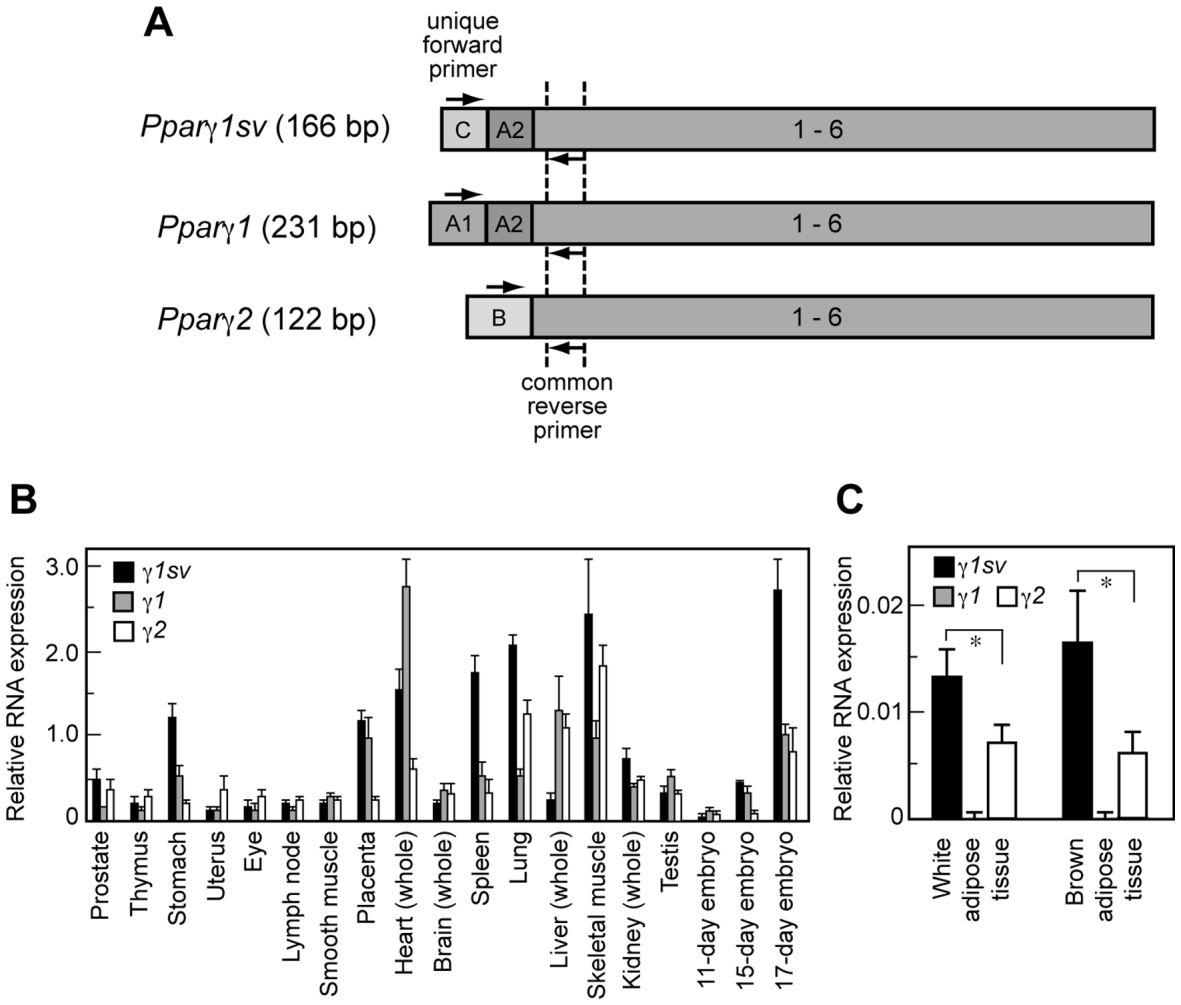
Tissue distribution and relative abundance of three *Pparγ* transcripts. (A) Schematic representation of three mouse *Pparγ* cDNAs. The positions of unique forward primers for each transcript and common reverse primer binding sites for qPCR analyses are indicated as arrows. The size of each PCR product is indicated in parenthesis. (B) Expression levels of *Pparγ1sv*, *Pparγ1*, and *Pparγ2* transcripts in mouse tissues were evaluated by qPCR analysis using the normalized first strand cDNAs as a template. The values represent the mean with error bars of triplicate measurements. (C) Expression levels of *Pparγ1sv*, *Pparγ1*, and *Pparγ2* transcripts in mouse white adipose tissue and brown adipose tissue. The values are normalized to the amount of 18S ribosomal RNA. The column denotes the data mean obtained from tissues of four animals. *P<0.01.

We designed unique forward primers for *Pparγ1sv*, *Pparγ1*, and *Pparγ2*, respectively and a common reverse primer for all *Pparγ* transcripts on exon 1 to quantify their expression levels (Fig. 2A). The relative expression levels of the three transcripts were analyzed by qPCR using normalized cDNAs prepared from 16 mouse tissues and embyos (Fig. 2B). *Pparγ1sv* was expressed abundantly in the stomach, placenta, heart, spleen, lung, skeletal muscle, and 17-day mouse embryo. *Pparγ1sv* was also abundantly expressed in the white and brown adipose tissues at higher levels than that of *Pparγ2* (Fig. 2C).

### Kinetics of *Pparγ1sv* Expression during Adipocyte Differentiation of 3T3-L1 and Primary Cells

**Figure 3:**
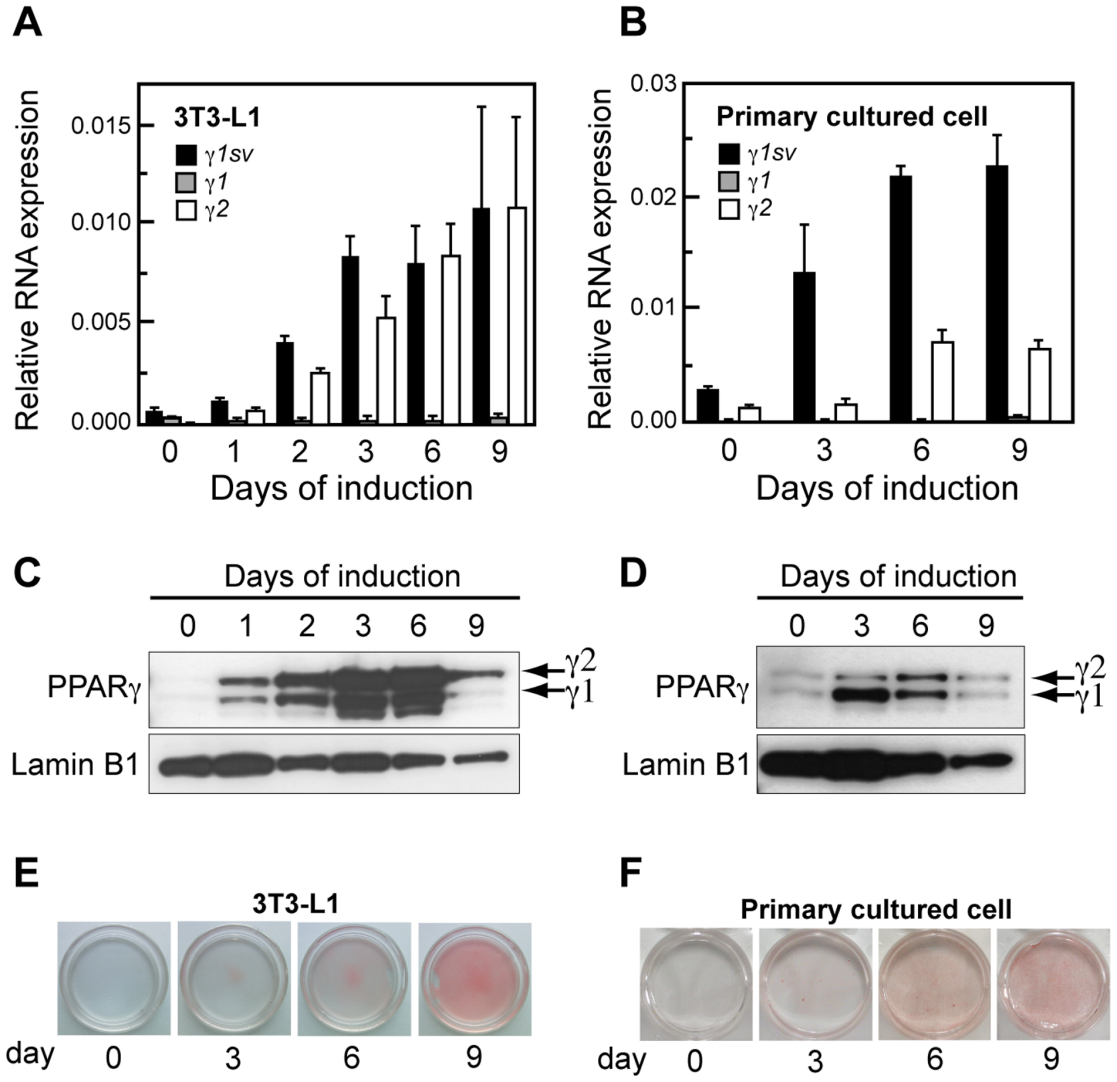
Expression of three *Pparγ* transcripts during adipogenesis of 3T3-L1 cells and mouse primary preadipocytes. 3T3-L1 cells (A) and mouse primary preadipocytes (B) were cultured in the differentiation medium described in the “Materials and Methods”, harvested at the indicated periods, and analyzed by real-time RT-PCR. The values obtained for *Pparγ1sv*, *Pparγ1*, and *Pparγ2* were normalized to those of 18S rRNA. Data represent the mean obtained from cells of three independent wells. Immunoblotting of nuclear extract of 3T3-L1 (C) and primary cultured cells (D) during adipocyte differentiation using the anti-PPARγ and anti-Lamin B1 (loading control) antibodies. Oil Red O staining of 3T3-L1 (E) and primary cultured cells (F) at indicated periods of adipocyte induction.

To further clarify the involvement of *Pparγ1sv* in adipogenesis, we examined its expression and kinetics in 3T3-L1 and primary cells from white adipose tissue of newborn mice during adipocyte differentiation. Both *Pparγ1sv* and *Pparγ2* mRNAs were induced in the early phase (day 1) of adipocyte differentiation of 3T3-L1 cells, and continued to increase up to day 9 (Fig. 3A). *Pparγ1sv* and *Pparγ2* mRNA levels were approximately 15- and 234-fold higher at day 9, respectively, than those of cells at day 0 (Fig. 3A). In primary cultured cells, the kinetics of *Pparγ1sv* and *Pparγ2* induction were similar to those of 3T3-L1 cells. The expression of *Pparγ1sv* and *Pparγ2* significantly increased upon differentiation up to day 6, and reached a plateau at day 9, respectively (Fig. 3B). No appreciable induction of *Pparγ1* mRNA was observed in the course of adipocyte differentiation of both 3T3-L1 and primary cells.

Immunoblotting of 3T3-L1 (Fig. 3C) and primary cultured cells (Fig. 3D) with anti-PPARγ antibody revealed that the PPARγ1 protein was abundant at day 3, expressed at day 6 with similar amount to the PPARγ2 protein, but reduced at day 9. We confirmed adipocyte differentiation of both 3T3-L1 (Fig. 3E) and primary cultured cells (Fig. 3F) by staining intracellular lipid accumulation with Oil Red O.

In ST2 cells, the kinetics of *Pparγ1sv* and *Pparγ2* induction were different from 3T3-L1 cells. The expression of *Pparγ1sv* and *Pparγ2* slightly increased upon differentiation but was down-regulated at days 6 and 9, respectively (Fig. S1A). This is probably due to the lower extent of adipocyte differentiation of ST2 cells. Upon stimulation with bone morphogenetic proteins, ST2 cells alternatively differentiate into osteoblasts. To assess if the up-regulation of *Pparγ1sv* is specific to adipogenesis in ST2 cells, we examined the expression level of *Pparγ1sv* in the course of osteoblast differentiation of ST2 cells. Expression levels of both *Pparγ1sv* and *Pparγ2* mRNAs were low and not markedly changed during differentiation (Fig. S1B). Alkaline phosphatase staining showed an increase in alkaline phosphatase activity, a hallmark of osteoblastic differentiation, in ST2 cells at 9 days after induction (Fig. S1B, inset photos).

### *Pparγ1sv* is Indispensable for Adipogenesis in 3T3-L1

**Figure 4:**
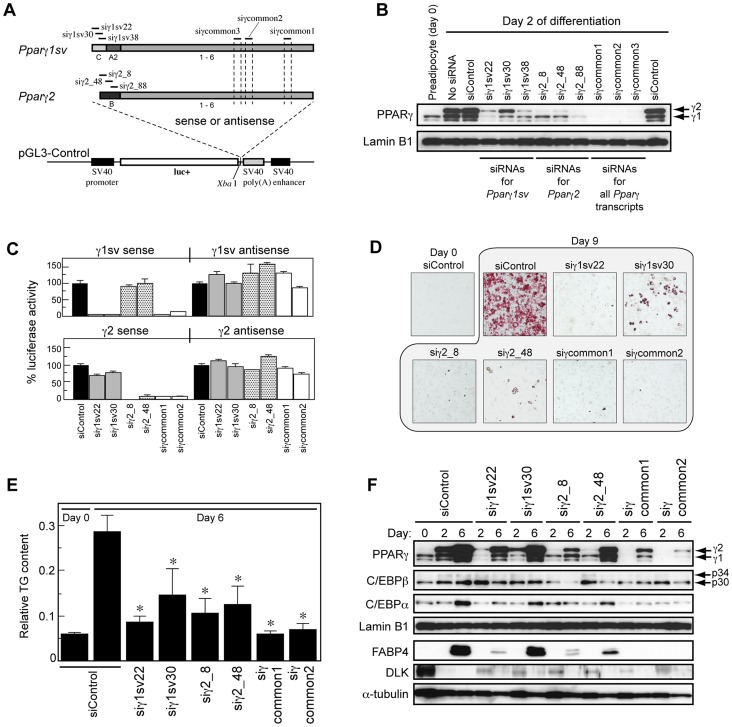
Effect of knock-down of *Pparγ1sv* on adipogenesis of 3T3-L1 cells. (A) Locations of the targeting sites of siRNAs for *Pparγ1sv*, *Pparγ2*, and both transcripts (common). For the validation of siRNAs using a luciferase reporter gene, the full-length *Pparγ1sv* or *Pparγ2* cDNA was inserted into *Xho* I site in pGL3-Control in sense or antisense direction. (B) Evaluation of siRNAs for *Pparγ1sv* or *Pparγ2*. 3T3-L1 cells were transfected with each of siRNA, and subjected to adipogenic induction in the following day (day 0). Expression of PPARγ1 and PPARγ2 proteins at day 2 was detected using PPARγ antibody with Lamin B1 as a control for nuclear extracts. siControl is negative control siRNA. Arrows indicate the positions of PPARγ1 and PPARγ2 proteins. (C) Validation of siRNA specificity. The relative luciferase activities were obtained from 3T3-L1 cells 1 day after transfection with pGL3-Control containing the *Pparγ1sv* or *Pparγ2* cDNA in sense or antisense direction and each of two respective siRNAs for *Pparγ1sv* and *Pparγ2*. The values represent the mean of triplicate measurements. The activity of siControl cells is defined as 100%. (D) 3T3-L1 cells transfected with siRNAs were subjected to adipogenic induction and stained by Oil Red O at day 9. Microscopic (x100) observations are shown. (E) Quantitative measurement of intracellular triglycerides in siRNA knock-down cells at days 0 and 6 using AdipoRed reagent. Data represent the mean of 4 replicate assays. *P<0.01 compared to siControl cells at day 6. (F) Protein expression levels of PPARγ, C/EBPβ, C/EBPα, Lamin B1 (control for nuclear extracts), FABP4 (aP2), DLK (pref-1), and α-tubulin (control for cytosolic extracts) in *Pparγ1sv* or *Pparγ2* knock-down cells at days 2 and 6 detected by the corresponding antibodies.

To evaluate whether the expression of *Pparγ1sv* is essential for the adipogenesis, we specifically knocked down *Pparγ1sv* mRNA in the early phase of the differentiation. We designed three specific siRNAs for respective targets, *Pparγ1sv* (siγ1sv22, siγ1sv30, and siγ1sv38) and *Pparγ2* (siγ2_8, siγ2_48, and siγ2_88) mRNAs. Positions of target sequences for designed siRNAs were indicated in Fig. 4A. 3T3-L1 cells were transfected with either of the siRNAs, and subjected to adipogenic induction in the following day. PPARγ1 and PPARγ2 protein levels were examined 2 days after induction by Western blotting using anti-PPARγ antibody (Fig. 4B). Introduction of all siRNAs for *Pparγ1sv* greatly reduced PPARγ1 protein levels relative to differentiated 3T3-L1 cells transfected with negative control siRNA (siControl in Fig. 4B). This indicates that most PPARγ1 protein was originated from *Pparγ1sv* mRNA in 3T3-L1 cells during adipocyte differentiation. In contrast, introduction of siRNAs for *Pparγ2* significantly suppressed PPARγ2 proteins at day 2. We also confirmed effective knock-down of both PPARγ1 and PPARγ2 proteins by introducing siRNA for the common region of the *Pparγ* coding sequence (siγcommon in Figs. 4A and 4B). In *Pparγ1sv* knock-down cells, PPARγ2 protein levels were notably reduced along with PPARγ1 proteins compared with siControl cells (Fig. 4B). Similarly, PPARγ1 proteins were partially reduced in *Pparγ2* knock-down cells (Fig. 4B). These results prompted us to evaluate specificity of these siRNAs in quantitative method. For this purpose, we used the luciferase-based reporter system, in which the full-length *Pparγ1sv* or *Pparγ2* cDNA was linked to luciferase gene (luc+) in sense or antisense direction (Fig. 4A). We excluded siγ1sv38 and siγ2_88 siRNAs from this validation assay because they showed less specificity in the knock-down of PPARγ1 and PPARγ2 proteins. In Fig. 4C, siγ1sv22 and siγ1sv30 siRNAs for *Pparγ1sv* achieved more than 95% knock-down of the reporter gene with the *Pparγ1sv* cDNA in sense direction (left upper panel) whereas they had negligible effect on the activity of the reporter with the *Pparγ2* cDNA in sense direction (left lower panel). On the other hand, siγ2_8 and siγ2_48 siRNAs for *Pparγ2* significantly reduced the activity of the reporter with *Pparγ2* cDNA (Fig. 4C, left lower panel) whereas no or little effect on the reporter with the *Pparγ1sv* cDNA (left upper panel). Both siγcommon siRNAs effectively knocked down the expression of the reporter gene with *Pparγ1sv* or *Pparγ2* in sense direction (Fig. 4C, left upper and left lower panel). Neither of siRNAs affected the activity of the reporter with *Pparγ1sv* or *Pparγ2* in antisense direction (Fig. 4C, right upper and right lower panel). These results confirmed that each siRNA could suppress its target mRNA with high specificity. We thus concluded that depletion of one PPARγ isoform affect the other’s protein level in 3T3-L1 cells during adipocyte differentiation.

We next examined the effect of knock-down of *Pparγ1sv* mRNA on the adipogenesis by Oil Red O staining at day 9 (Fig. 4D) and quantitation of intracellular triglycerides at day 6 (Fig. 4E). Both results showed that knock-down of *Pparγ1sv* completely (by siγ1sv22) or substantially (by siγ1sv30) inhibited the lipid accumulation as observed in *Pparγ2* knock-down cells, implying the importance of *Pparγ1sv* in adipogenesis. We further characterized siγ1sv- and siγ2-transfected cells by analyzing the expression of the adipocyte-related proteins C/EBPα, C/EBPβ, FABP4 (aP2), and DLK (pref-1) by Western blotting (Fig. 4F). Transfection of siγ1sv22 or siγ1sv30 resulted in no apparent change in protein levels of C/EBPβ at days 2 and 6 compared with those of the control cells whereas a slight inhibition in protein levels of C/EBPα at day 6. Induction of FABP4, an adipogenic marker protein, was markedly inhibited in siγ1sv22 and siγ2_8 cells at day 6, but neither in siControl nor in siγ1sv30 cells. The expression of DLK, a preadipocyte marker, was drastically down-regulated upon differentiation in siControl cells. It was also decreased but could be detected at a very low level in all siγ1sv, siγ2, and siγcommon knock-down cells at day 2 (Fig. 4F).

### *Pparγ1sv* Expression is Dependent on C/EBPβ and C/EBPδ in 3T3-L1 Cells

**Figure 5:**
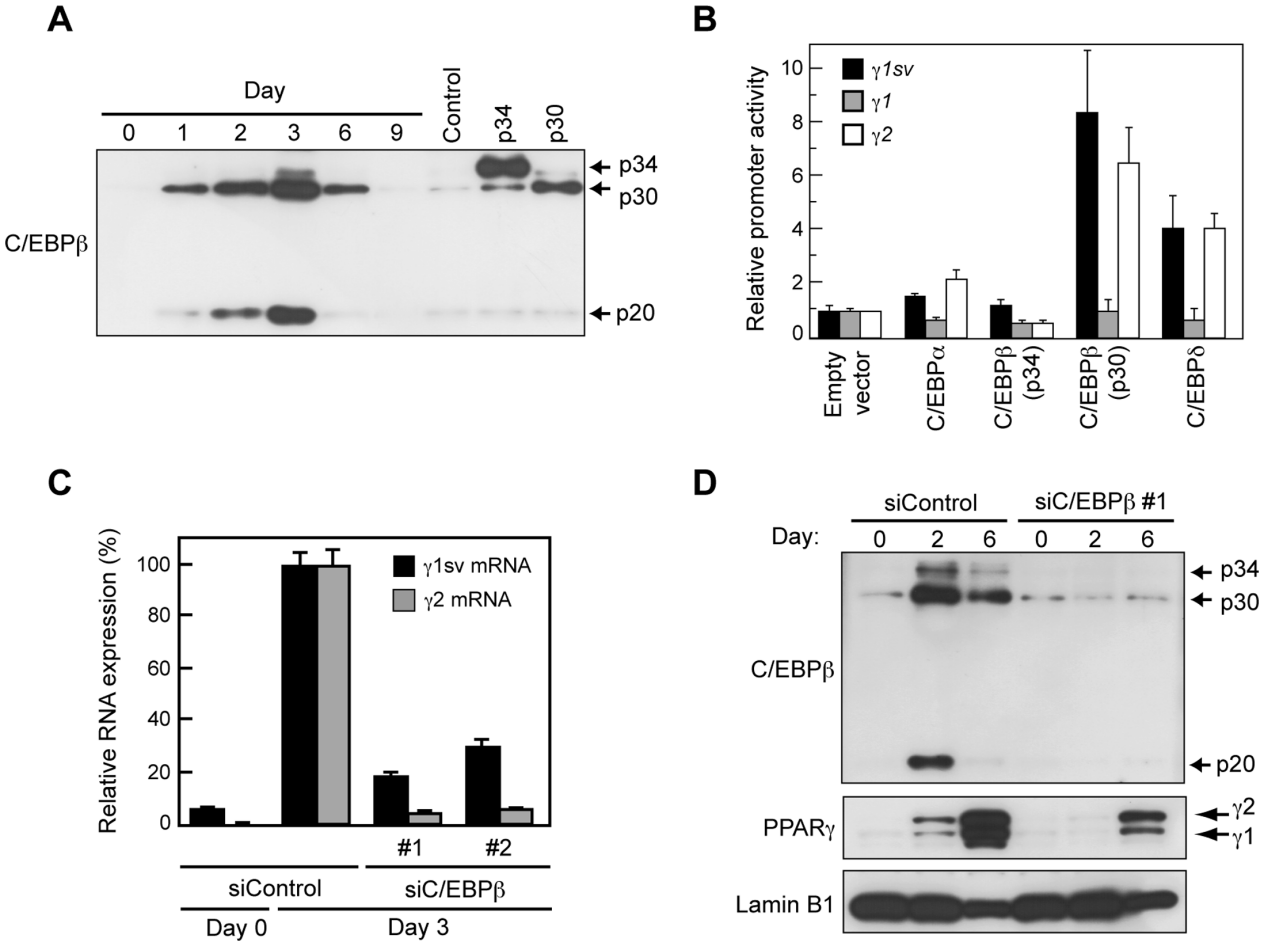
Luciferase reporter assay for the *Pparγ1sv* promoter in 3T3-L1 cells. (A) Western blotting of 3T3-L1 cells during adipocyte differentiation (days 0–9) and NIH/3T3 cells transfected with the overexpression construct containing no insert (control) or coding region of C/EBPβ isoforms (p34 and p30) detected by anti-C/EBPβ antibody. (B) The luciferase reporter construct, the pGL3-Basic, containing the *Pparγ1sv*, *Pparγ1*, or *Pparγ2* promoter was co-transfected with the C/EBP overexpression construct to 3T3-L1 cells. Cells were harvested 2 days after transfection and assayed using Dual-luciferase reporter assay reagents. The values represent the mean of triplicate measurements. The activity obtained from cells transfected with empty vector is defined as 1. (C) Effect of C/EBPβ depletion by siRNA on *Pparγ* expression. Each of two discrete C/EBPβ siRNAs (siC/EBPβ #1 and #2) was transfected to 3T3-L1 cells. Cells were harvested at days 0 and 3 of differentiation, and expression of *Pparγ1sv* (black bar) and *Pparγ2* (gray bar) mRNAs was evaluated by qPCR. Values were normalized to those of 18S rRNA. The RNA expression of siControl cells at day 3 is defined as 100%. (D) Immunoblotting of the nuclear extracts of siC/EBPβ #1-treated cells detected by antibodies specific to each of C/EBPβ, PPARγ, or Lamin B1 (control).

The significant induction of mouse *Pparγ1sv* mRNA during adipocyte differentiation raised a question of how transcription of *Pparγ1sv* is regulated. C/EBPβ and C/EBPδ are induced within a day during adipogenesis of 3T3-L1 cells [4]. This in turn activates expression of *Pparγ* and C/EBPα. The two reciprocally stimulate each other by forming a positive feedback loop, and synergistically promote the downstream gene expression required to accomplish adipogenesis. The promoter of *Pparγ2* contains two C/EBP recognition elements, and *Pparγ2* is directly up-regulated by C/EBPα and C/EBPδ [14]. To clarify whether C/EBPs up-regulate *Pparγ1sv* as well, we performed a luciferase reporter assay using the *Pparγ1sv* promoter (−969 to +50) that had been subcloned into the luciferase reporter vector pGL3-Basic. The reporter construct was co-transfected with the expression vector harboring either of the coding sequence of C/EBPα, C/EBPβ, or C/EBPδ. C/EBPα and C/EBPβ have several isoforms, which include full-length and N-terminally truncated proteins [15]. In differentiating 3T3-L1 cells, we detected three C/EBPβ isoforms, full-length (p34) and two N-terminally truncated C/EBPβ proteins (p30 and p20). Of the three, C/EBPβ (p30) was the dominant isoform (Fig. 5A). As shown in Fig. 5B, C/EBPβ (p30) and C/EBPδ markedly increased the promoter activities of *Pparγ1sv* and *Pparγ2*, while full-length C/EBPβ (p34) did not. Overexpression of C/EBPα gave a slight but significant increment (*P*<0.05) in both *Pparγ1sv* and *Pparγ2* promoter activities compared to control cells, respectively. *Pparγ1* promoter activity was not altered by co-transfection with each of the overexpression vectors (Fig. 5B). We next examined the effect of C/EBPβ knock-down on the expression levels of *Pparγ1sv* and *Pparγ2* mRNAs after the induction of adipocyte differentiation. Each of two discrete siRNAs targeting C/EBPβ were transfected into 3T3-L1 cells. As shown in Fig. 5C, expression of both *Pparγ1sv* and *Pparγ2* mRNAs were markedly inhibited in C/EBPβ knock-down cells at day 3 of induction. Protein levels of PPARγ1 and PPARγ2 were also significantly suppressed at days 2 and 6 of induction by transfecting C/EBPβ siRNA (#1) relative to those of control siRNA (Fig. 5D).

## Discussion

In this study, we analyzed 5′-ends of mouse *Pparγ* cDNAs and isolated full-length cDNA of the novel splicing variant, *Pparγ1sv*, that encodes PPARγ1 protein. *Pparγ1sv* was remarkably up-regulated with an induced adipogenesis. Knock-down of *Pparγ1sv* in adipocytes resulted in the substantial reduction of the PPARγ1 protein and intracellular lipid accumulation, indicating an indispensable role of *Pparγ1sv* in adipocyte differentiation.

Several *Pparγ* splicing variants except for *Pparγ1* and *Pparγ2* have been identified in humans [12], [13], [16]–[18], monkeys [19], and pigs [11]. Mouse *Pparγ1sv* and corresponding splicing variants in the above 3 mammals and rats share a unique exon (named C in mouse) (Table 1), which implies that the expression of this splicing variant is ubiquitous in mammals.

The expression profiling of three *Pparγ* transcripts showed that their different abundance in mouse tissues (Figs. 2B and 2C). In several tissues, *Pparγ1sv* is expressed at higher levels than the others. For example, expression level of *Pparγ1sv* in spleen was 3.3 and 5.3 times higher than those of *Pparγ1* and *Pparγ2*, respectively. Thus, *Pparγ1sv* could be a major transcript and contribute to the PPARγ protein expression the most in those tissues. While localization of *Pparγ1sv* in mouse embryo is undetermined, *Pparγ1sv* is dramatically up-regulated during the late stages of fatal development (15- and 17-day, Fig. 2B), implying that *Pparγ1sv* is deeply involved in cell differentiation in embryo. The pathological analyses of PPARγ-deficient mice revealed PPARγ functions in multiple tissues such as the adipose tissue, the placenta, and the developing heart during pre- and postnatal development [20]. Recently, overlapping and distinct functions of PPARγ1 and PPARγ2 in prostate epithelial cells have been reported [21]. We are presently generating *Pparγ1sv*- and/or *Pparγ1*-deficient mouse to assess the specific roles of each isoform in development, which will provide some answers to the meaning and importance of the production of multiple transcripts in *Pparγ*.

To date, PPARγ2 protein but not PPARγ1 is thought to play an essential role in adipogenesis, because *Pparγ2* mRNA is up-regulated during the initiation of adipocyte differentiation whereas *Pparγ1* is not. In this study, we showed that *Pparγ1sv* is highly expressed in the white and brown adipose tissues (Fig. 2C), which indicates considerable expression of not only PPARγ2 but also PPARγ1 protein in the adipose tissues. In fact, both PPARγ1 and PPARγ2 proteins drastically increased during adipogenesis of 3T3-L1 and primary cultured cells (Figs. 3C and 3D). We showed that *Pparγ1sv* is markedly up-regulated during adipocyte differentiation (Figs. 3A and 3B). The knock-down of *Pparγ1sv* using siRNAs resulted in significant suppression of PPARγ1 protein during adipocyte differentiation of 3T3-L1 cells (Fig. 4B). These results strongly support that PPARγ1 protein expressed during adipogenesis is derived from *Pparγ1sv* mRNA. Knock-down of *Pparγ1sv* also greatly inhibited the accumulation of intracellular triglyceride (Fig. 4E) and the induction of an adipocyte marker FABP4 (siγ1sv22 in Fig. 4F) in 3T3-L1 cells. Incomplete adipocyte differentiation of siγ1sv- and siγ2-transfected cells was also confirmed by the partial expression of a preadipocyte marker, DLK at day 2 (Fig. 4F). It was likely that inhibition of adipocyte differentiation evaluated by lipid accumulation and marker proteins was dependent on the abundance of PPARγ1 and PPARγ2 proteins in siγ1sv- and siγ2-treated cells. We thus concluded that the up-regulation of PPARγ1 proteins originated from *Pparγ1sv* during adipogenesis is indispensable to accomplish the differentiation process.

C/EBPβ and C/EBPδ play key roles in the early phase of the adipogenic molecular cascade. Expression of both proteins is enhanced during the initial few hours of differentiation in 3T3-L1, which in turn activate expression of *Pparγ2* and C/EBPα. We have found that C/EBPβ and/or C/EBPδ also activated the *Pparγ1sv* promoter (Fig. 5B). Unexpectedly, N-terminally truncated C/EBPβ (p30) significantly activated the both *Pparγ1sv* and *Pparγ2* promoters (Fig. 5B), but full-length C/EBPβ isoform (p34) did not. We demonstrated that the major product in adipogenesis of 3T3-L1 cells was p30 (Fig. 5A). Therefore, it is possible that p30 and C/EBPδ directly initiate the synergistic expression of *Pparγ1sv* and *Pparγ2* mRNA in the early period of adipogenesis.

**Figure 6:**
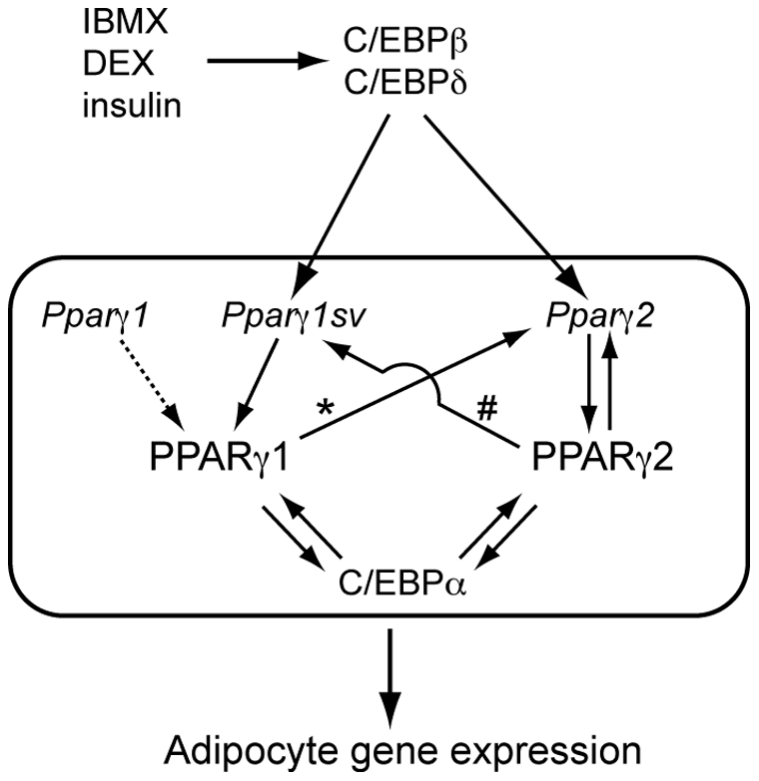
Schematic model for the transcriptional control of *Pparγ* in adipocyte differentiation. C/EBPβ and C/EBPδ directly transactivate both *Pparγ1sv* and *Pparγ2* genes, which are in turn translated to PPARγ1 and PPARγ2 proteins, respectively. PPARγ ensures the expression of downstream genes involved in adipogenesis, with forming a positive feedback loop with C/EBPα. Arrows with an asterisk and a sharp are speculative feedback pathways for *Pparγ* up-regulation by PPARγ proteins.

Intriguingly, inhibition of either of *Pparγ* transcript by specific siRNA resulted in suppression of both PPARγ proteins (Fig. 4B). Validation of designed siRNAs using the luciferase reporter system showed their highly effective and specific knock-down properties (Fig. 4C). These results imply that expression level of PPARγ1 protein could affect that of PPARγ2 and vice versa during adipogenesis of 3T3-L1 cells. One possible explanation is the direct up-regulation of the *Pparγ* transcription by PPARγ proteins. It has been demonstrated that *Pparγ2* gene expression is regulated by binding of the PPARγ/RXRα heterodimer to the *Pparγ2* promoter during adipocyte differentiation of 3T3-L1 [8]. Although direct interaction of the PPARγ1 protein to the *Pparγ2* promoter has not been clarified, it is probable that depletion of the PPARγ1 protein by siRNA targeting to *Pparγ1sv* caused reduction in the amount of the PPARγ/RXRα heterodimer, which resulted in less activation of the *Pparγ2* promoter and down-regulation of the PPARγ2 protein (Fig. 6, arrow with an asterisk). On the other hand, binding of the PPARγ/RXRα heterodimer to the regions of the *Pparγ1sv* promoter (Fig. 6, arrow with a sharp) was not observed [8]. We could not identify the consensus sequence for PPARγ and RXRα binding in the *Pparγ1sv* promoter (∼1 kb). Therefore, down-regulation of *Pparγ1sv* by the introduction of *Pparγ2*-specific siRNA might be involved in downstream factors that are regulated by PPARγ2 protein and activate the *Pparγ1* promoter.

The present study suggests the importance of *Pparγ1sv* in the adipocyte differentiation and a real need to elucidate a detailed mechanism of the *Pparγ1sv* regulation and the precise function of PPARγ1 protein in cell differentiation.

## Supporting Information

Figure S1 **Relative expression of three PPARγ transcripts during adipocytic and osteoblastic differentiation of ST2 cells.** (A) Confluent ST2 cells were cultured in RPMI1640 medium supplemented with 10% FBS, 0.25 µM dexamethasone, 500 µM isobutylmethylxanthine, 1 µM insulin, and 1 µM rosiglitazone to induce adipocytic differentiation. Cells were harvested at the indicated time and analyzed by real-time RT-PCR. (B) ST2 cells were cultured in RPMI1640 medium supplemented with 10% FBS and 100 ng/ml BMP-4 (Wako, Japan) to induce osteoblastic differentiation. Cells were analyzed by real-time RT-PCR or fixed with 10% formalin for 20 min and stained using an alkaline phosphatase staining kit (Primary Cell Co., Ltd).
(TIF)
