# Supplementary figures and images for: A Novel Splicing Variant of Peroxisome Proliferator-Activated Receptor-γ (Pparγ1sv) Cooperatively Regulates Adipocyte Differentiation with Pparγ2
Source: PLoS One. 2013 Jun 19;8(6):e65583. doi: 10.1371/journal.pone.0065583 (PMC3686765; doi:10.1371/journal.pone.0065583)

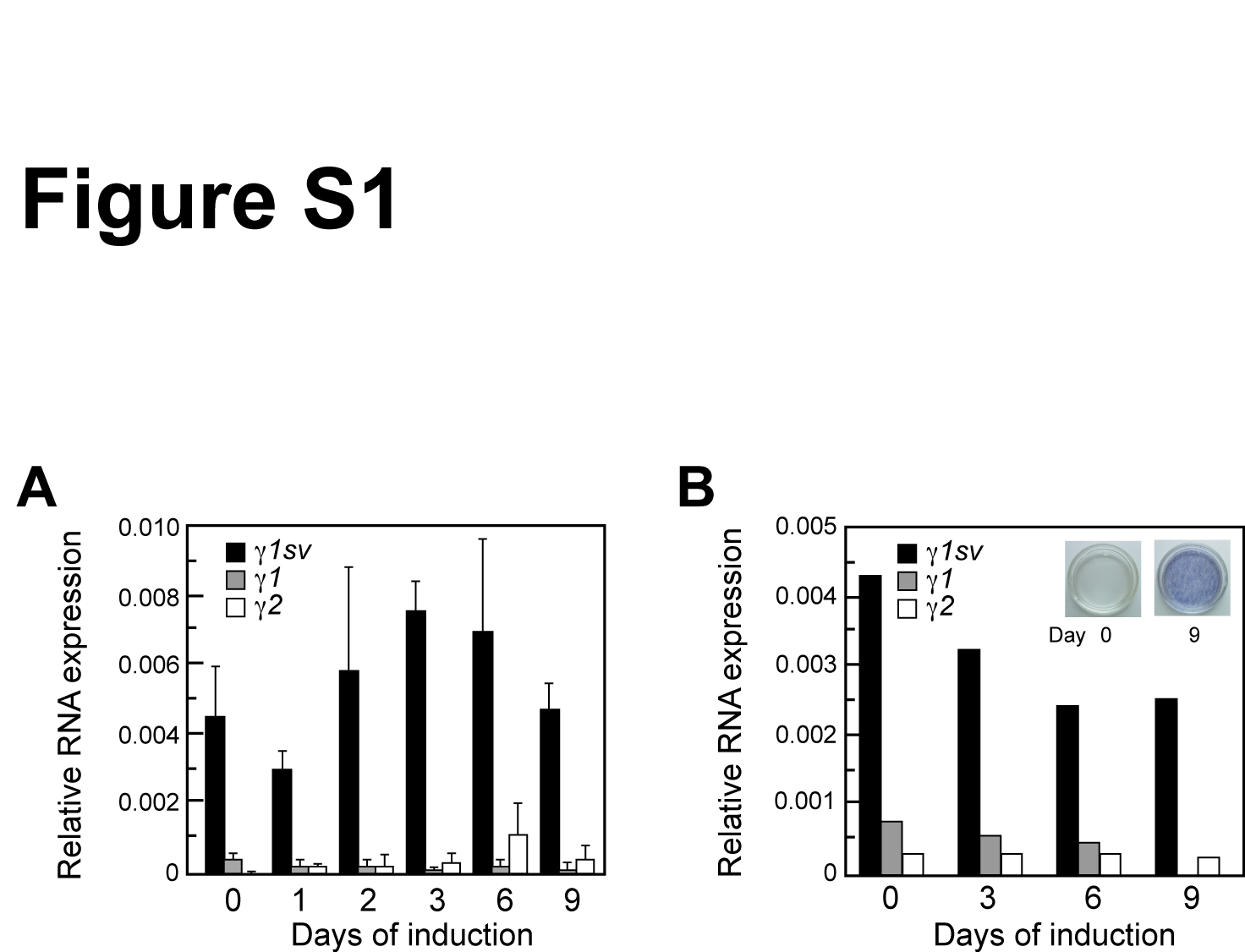

Supplement: Figure S1 — Relative expression of three PPARγ transcripts during adipocytic and osteoblastic differentiation of ST2 cells. (A) Confluent ST2 cells were cultured in RPMI1640 medium supplemented with 10% FBS, 0.25 µM dexamethasone, 500 µM isobutylmethylxanthine, 1 µM insulin, and 1 µM rosiglitazone to induce adipocytic differentiation. Cells were harvested at the indicated time and analyzed by real-time RT-PCR. (B) ST2 cells were cultured in RPMI1640 medium supplemented with 10% FBS and 100 ng/ml BMP-4 (Wako, Japan) to induce osteoblastic differentiation. Cells were analyzed by real-time RT-PCR or fixed with 10% formalin for 20 min and stained using an alkaline phosphatase staining kit (Primary Cell Co., Ltd). (TIF) [file pone.0065583.s001.tif]
